# Temporal Dynamics of Species Richness and Composition in a Peri‐Urban Tropical Frog Community in Central Brazil

**DOI:** 10.1002/ece3.70628

**Published:** 2024-11-25

**Authors:** Marcos R. Severgnini, Mônica M. de Oliveira, Luciana M. Valério, Diogo B. Provete

**Affiliations:** ^1^ Graduate Program in Ecology and Conservation, Institute of Biosciences Federal University of Mato Grosso Do Sul Campo Grande Mato Grosso do Sul Brazil; ^2^ Fragment Ecology Study Group Campo Grande Mato Grosso do Sul Brazil; ^3^ Institute of Biosciences Federal University of Mato Grosso Do Sul Campo Grande Mato Grosso do Sul Brazil; ^4^ Catholic University Dom Bosco Campo Grande Mato Grosso do Sul Brazil; ^5^ Gothenburg Global Biodiversity Centre Göteborg Sweden; ^6^ German Centre for Integrative Biodiversity Research –iDiv Halle‐Jena‐Leipzig Leipzig Germany

**Keywords:** beta diversity, Cerrado, phenology, species‐time relationship, time series

## Abstract

Analyzing the temporal dynamics of ecological communities can shed light on coexistence mechanisms and help understand how populations and communities will behave in the face of climate change. However, little is known about how frog communities respond to climate in urban ecosystems, especially in tropical countries. Here, we analyzed how frog species richness and abundance are influenced by weather variables both intra‐ and inter‐annually. We surveyed a peri‐urban area in central Brazil, monthly for 3 years. To test the effect of weather variables on species richness and abundance, we used Generalized Additive Mixed‐effects Models. We assessed seasonality using circular statistics. We also tested for differences in temporal beta diversity within and among years by estimating species disappearance and temporal rank shift, in addition to a multivariate model‐based method to test the effect of year on species composition. Finally, we tested how taxonomic and phylogenetic alpha diversity changed through time using a novel approach based on Hill numbers. We found that species richness varied little among years and was affected only by photoperiod, while species abundance was more variable both between and within years, being mostly affected by humidity, temperature, and photoperiod. Species composition varied little between years, mostly between the first and subsequent years. Conversely, beta diversity was highest within years. Only the effective number of species changed significantly through time. Our results help not only understand temporal mechanisms that allow species coexistence, but also allow to make inferences about the impact of urbanization on biodiversity in recently urbanized landscapes, showing that species composition in peri‐urban sites remains unaltered in a mid‐timescale, especially when climate conditions change little across years.

## Graphical abstract

This study address how species coexistence and species–environment relationship are impacted by temporal variation in climate sub‐urban area.

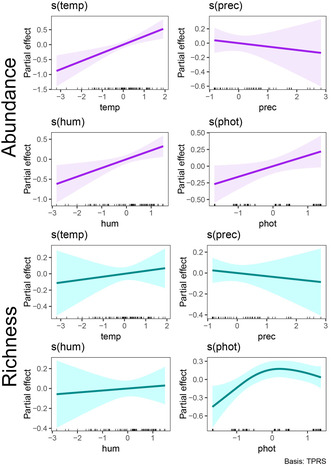

## 1 Introduction

Analyzing temporal dynamics allows to forecast changes in the long‐term structure of ecological communities (Dornelas et al. 2013). Time is a key niche axis influencing patterns at population and community scale (Post 2019). Long‐term studies allow us to identify how the environment is changing, and which strategies species adopt to survive. Species with different life‐history strategies can use resources in different ways at the same time, allowing them to co‐exist (Tokeshi 1999; Post 2019; Rudolf 2019). For instance, some frog species might advance their breeding season and lay eggs earlier than others to avoid competition for breeding sites. This is the case of *Rhinella diptycha* and *Leptodactylus macrosternum* that breed in the Pantanal during the autumn—winter, while most species breed during the summer (Prado, Uetanabaro, and Haddad 2005). This strategy allows their tadpoles to grow as fast as possible avoiding interspecific competition with later arriving species (Buxton and Sperry 2016). However, most studies assessing the temporal distribution of frog species in the tropics were either short termed (small extent, e.g., Aichinger 1987) or had sparse samplings (broad scale).

Abiotic factors can vary through time both within (e.g., seasons) and between years. Consequently, species use abiotic factors as environmental cues to track favorable conditions (Post 2019), and individuals that best recognize them increase their survival and reproductive rates in the long term. Environmental cues, such as weather (e.g., rainfall, temperature, and humidity) or astronomic variables (e.g., photoperiod) are well known to modulate reproductive phenology and life‐story traits (Post 2019) in several taxonomic groups, such as reptiles (Brown and Shine 2002), birds (Walther et al. 2017), mammals (Appel et al. 2019), and especially amphibians (Canavero et al. 2008, 2019; Canavero and Arim 2009; Ceron et al. 2020; Smaniotto and Moreira 2023; Neptune and Benard 2023). In tropical seasonal environments, rainfall and humidity are more important than temperature and photoperiod to determine anuran occurrence and calling activity (Canavero, Arim, and Brazeiro 2009; Ceron et al. 2020). Conversely, photoperiod is more often used by anuran species to predict favorable breeding conditions in temperate and subtropical environments (Both et al. 2008; Canavero and Arim 2009).

Quantifying temporal changes in biodiversity is challenging (Dornelas et al. 2013), since long‐term studies require more resources and collaboration among researchers. However, differently from most studies that evaluate only 1 year and take a “snapshot” of the temporal process, mid‐term and long‐term studies can shed light on ecological and evolutionary processes acting at the community scale (Gomes‐Mello et al. 2021). Several processes might act at different timescales, such as sampling effect, ecological and evolutionary processes (Rosenzweig 1995; Korhonen, Soininen, and Hillebrand 2010). However, both deterministic processes, such as species sorting (Leibold and Chase 2018) that select species that best fit environmental conditions, and demographic stochasticity (Chase 2010) can act over short, mid, or long timescales to determine patterns at the community scale. Nonetheless, long‐term data from diverse, tropical environments are still scarce but urgently needed to test the generality of theoretical predictions.

Here, we evaluated the temporal changes in a peri‐urban frog community as driven by weather variables and astronomic (i.e., photoperiod) variables along ecological (i.e., seasonal) and cosmological time (i.e., among years). This is the first mid‐term study that evaluated compositional changes in a tropical peri‐urban frog community (reviewed in Severgnini et al. 2024), in addition to investigating temporal changes in taxonomic and phylogenetic alpha diversity. Additionally, we tested how intra‐ and interannual beta diversity and abundance rank shift vary. Finally, we tested how species richness and abundance of a frog community are influenced by weather variables for three consecutive years. Specifically, we asked the following questions: (1) Does taxonomic and phylogenetic alpha diversity change through time? (2) How seasonal are species richness and abundance within years? (3) How weather variables affect species richness and abundance both within and between years? and (4) How does temporal beta diversity, and the abundance rank of species, change within and between years?

## 2 Methods

### 2.1 Study Site and Sampling Design

**FIGURE 1:**
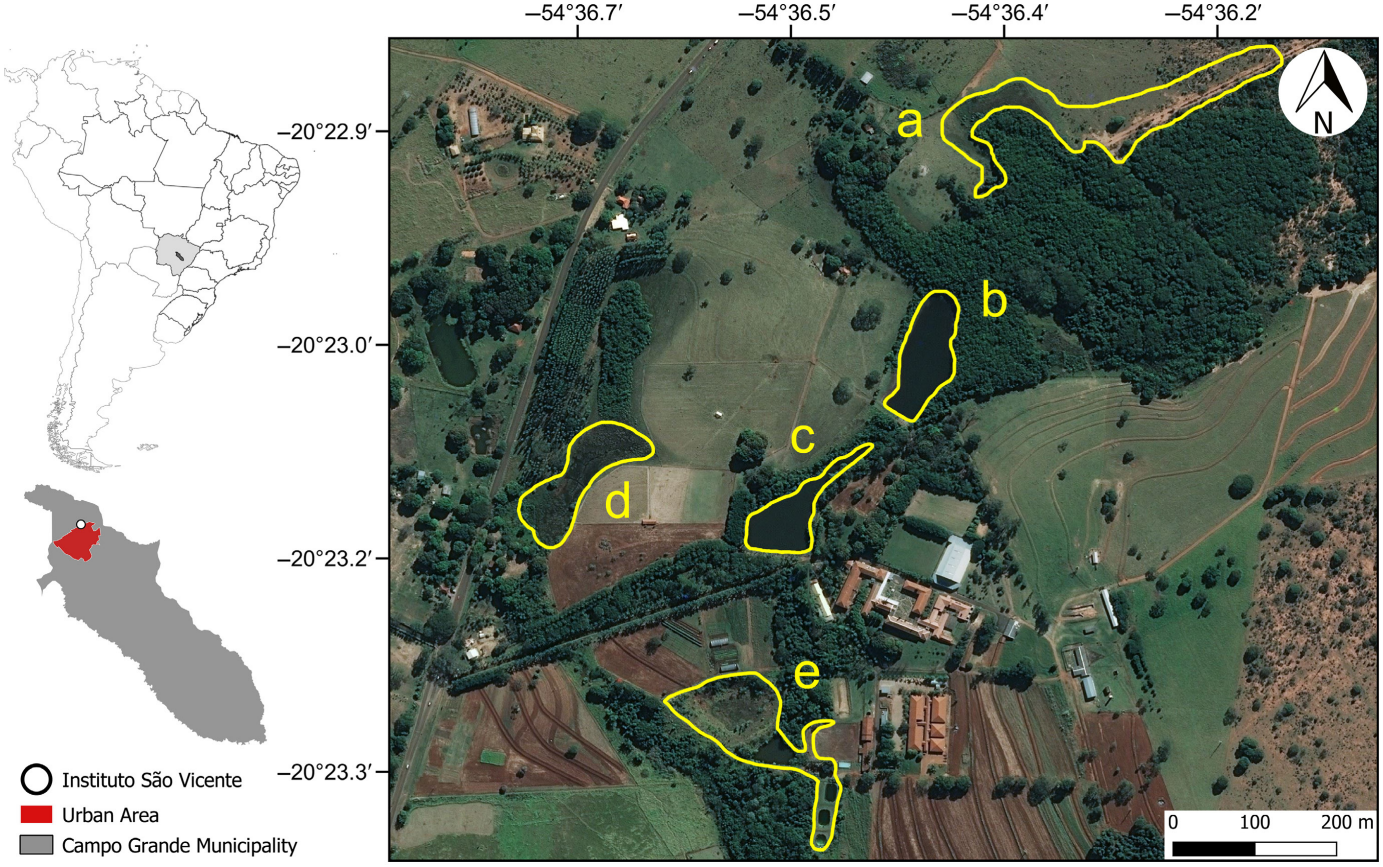
Map of the study site highlighting the temporary and permanent ponds surveyed for frogs: (a) Temporary Pond with wet soil trampled by cattle; (b) Man‐made permanent pond; (c) Man‐made permanent pond in open area; (d) Natural temporary pond; (e) Natural temporary pond. Map features extracted from Instituto Brasileiro de Geografia e Estatística (IBGE) and Google Earth; and prepared on QGIS v. 3.22.1.

We conducted fieldwork at the São Vicente Institute (20°23′03.6″ S; 54°36′26.9″ W; 529 m a.s.l.; DATUM = SIRGAS/2000), a peri‐urban area approximately 10 km away from the center of Campo Grande, Mato Grosso do Sul, central Brazil (Figure 1). The local climate is Equatorial Savanna or Köppen's Aw (Kottek et al. 2006), with two well‐defined seasons: a dry winter (June–September) and a rainy summer (December–March), and two variable seasons with relatively hot spring (October–November) and dry autumn (April–May). The mean temperature is 23.3°C, with mean annual precipitation around 1579.7 mm (Mendonça and Danni‐Oliveira 2007).

We surveyed frogs monthly from August 2014 to August 2017 in five ponds, on average 441 m apart from one another (range 245–812 m; Figures 1 and S1) using visual encounter surveys (Crump and Scott Jr. 1994). We walked around each pond in a one‐way direction looking for frogs on the margin, leaf litter, under the ground, floating vegetation, low grassy vegetation, and tree branches. Ponds were surrounded by similar land use types. For example, the land use in a 1‐km buffer around pond d (Figure 1) comprises 68.3% pasture, 7.69% forest, 4.8% soybean, 4.29% urban area, and 2.32% wetlands (data from MapBiomas 2023). Field campaigns lasted for two to three nights each month, during which we sampled one to three ponds each night. The surveys always started at 18:00 and lasted until right after midnight. Our sampling effort was 2 h per pond monthly, with an average of three people per night (by the same surveyors to ensure consistency of skill and standardization), for a total of 1080 h (hours*ponds*person*months*years). Most specimens seen or heard were captured and identified. We follow Frost (2023) for nomenclature. We did not collect specimens nor use mark recapture. Thus, our abundance estimates may be biased. However, we did not sum the abundance per month, but used the abundance of each sampling date (i.e., 79 sampling events) separately to perform statistical analysis. Furthermore, we lumped the abundance and richness for all five ponds to focus on temporal patterns. To ensure that the data did not have spatial autocorrelation, we performed a Mantel correlation between compositional and geographical distance matrix. We found a weak and non‐significant relationship (see Figure S2).

### 2.2 Weather Variables

We obtained daily data for temperature (°C), relative humidity (%), and rainfall (mm) from an automatic station ID‐A702 (13.9 km away from the study site) from the Brazilian National Meteorology Institute (INMET, n.d.) and photoperiod (minutes of daylight) from the Brazilian National Observatory (ON, n.d.). We took a 3‐day‐average (2 days prior each sampling and the day of sampling) from each variable. We standardized the data to zero mean and unit variance in the R package *vegan* (Oksanen et al. 2018) and checked for multicollinearity using Variance Inflation Factor (VIF) in the R package *usdm* (Naimi et al. 2014) in R software 4.2.3 (R Core Team 2023). All variables had VIF < 3 and were retained for further analysis.

### 2.3 Data Analysis

To estimate sampling completeness, we first built a sample completeness curve (Gotelli and Colwell 2001) with abundance data. Analysis was conducted in the R package *iNEXT* (Chao et al. 2014).

To estimate the diversity profile of the community, we performed interpolation and extrapolation using Hill numbers with three orders of the *q* exponent in the R package *iNEXT*. Hill numbers are a parametric family of diversity indices that provide an estimation of the effective number of species in an assemblage under a unified mathematical framework. The effective number of species is the “number of equally abundant species that are needed to give the same value of the diversity measure” (Chao et al. 2014). The index differs only by an exponent *q*, which weights differently species abundance and evenness, in a way that *q* = 0 is equivalent to species richness, *q* = 1 is equivalent to Shannon index or the effective number of common species, and *q* = 2 is equivalent to Gini‐Simpson index or the effective number of dominant species (Chao et al. 2014). Additionally, we estimated the temporal distribution of the taxonomic and phylogenetic alpha diversity using the *iNEXT.3D* package (Chao et al. 2021). This method allows estimating the asymptotic diversity profile, along with 95% CI for each *q* order, and comparing its temporal trend. By comparing the observed with the estimated 95% CI derived from the sample coverage, it allows testing for temporal change in each *q* order.

The phylogenetic tree for the community was obtained by pruning the fully‐sampled, Maximum Clade Credibility Tree of Jetz and Pyron (2018) for 28 species that occurred in the study site (Figure S3). This phylogenetic tree was built from a molecular backbone tree with 15 genes for 4061 amphibian species and then used taxonomic imputation to obtain a fully‐sampled tree for 7238 extant amphibian species (see Jetz and Pyron 2018).

To test for seasonality in species richness and abundance, we performed a circular analysis (Zar 2010) using ORIANA v. 4.02 (Kovach 2013). Months were converted into angles at 30° intervals, and richness and abundance converted into frequencies of each angle (months). Then, we calculated Rayleigh's *z* to test whether species abundance and richness were concentrated into certain months based on the length of the mean vector (*r*) (Zar 2010). This parametric test based on the von Mises distribution (i.e., normal distribution in circular data) allows estimating other parameters, including number of observations (*n*); mean angle (*α*), which represents the angle in which most species are concentrated; and the circular standard deviation (SD), which is related to the mean vector (Zar 2010).

To test whether weather variables (predictor variables) influenced observed species richness and abundance (response variables), we used Generalized Additive Mixed‐effects Models (GAMM), with Poisson error distribution for richness and negative binomial for abundance (Zuur et al. 2009; Wood 2017). Analysis was performed using the *mgcv* R package (Wood 2017). To control for temporal autocorrelation, we used an Auto Regressive Integrated Moving Average (ARIMA; Hyndman and Khandakar 2008). To select the best *p* and *q* parameters, we first fitted six different models for richness and abundance in the *nlme* package (Pinheiro et al. 2021), including year, month, and date. We built the corARMA formula specifying a time covariate and a grouping factor (e.g., ~1|month), where default ~1 is the observation order as a covariate, and date, month, or year as a grouping factor. This is adequate, because our data have two or three replicates per month over 3 years. Then, we used the Akaike Information Criteria (AICc; Burnham and Anderson 2002) in the *bbmle* R package (Bolker and R Development Core Team 2020) to rank models. Month was the best grouping factor for abundance, while year was best for richness. We also performed residual diagnostics using the autocorrelation function in the *stats* (R Core Team 2023) and *gratia* R packages (Simpson 2023). Furthermore, we checked for overdispersion using the *DHARMa* R package (Harti 2021).

To test for compositional variation through time, we used two complementary approaches: (i) From our species composition matrix, we calculated the total temporal turnover and decomposed it into disappearance and appearance of species over months to identify how species identity is changing; (ii) to test if species rank abundance patterns are changing within and between years, we calculated the mean rank shift in the *codyn* R package (Hallett et al. 2016). This analysis allows testing how the dominance pattern in the community changes over time, which can indicate changes in competitive hierarchy, for example, between seasons.

Finally, we used a model‐based approach to test for the variation in species composition across years. We fitted a model for multivariate abundance as a function of year using negative binomial distribution in the *mvabund* R package (Wang et al. 2012) and performed residual diagnostics using Dunn‐Smyth residual plots. Residuals had normal distribution and homogeneity of variance. Subsequently, we performed a post hoc test to analyze pairwise differences in compositional variation between years. Results were visualized using a model‐based, unconstrained ordination with two latent variables in the *ecoCopula* R package (Popovic, Hui, and Warton 2018). Also, to detect which species contributed the most for compositional variation among years, we performed univariate tests, adjusting *p* values for multiple testing. All data and associated R code are available at FigShare (Severgnini et al. 2023).

## 3 Results

We recorded 2162 individuals from 28 species across all ponds throughout 3 years (Figure S4; Tables S1 and S2). The five most abundant species were *Leptodactylus podicipinus* (*n* = 500), *Dendropsophus nanus* (*n* = 312), *Scinax fuscomarginatus* (*n* = 301), *Dendropsophus minutus* (*n* = 294), and *Boana punctata* (*n* = 217; Tables S1 and S2). The months with highest species richness were September, October, November, and February with a peak in February 2015 (*n* = 12). The ones with highest abundances were January, February, March, and April, with peak abundance in January 2017 (*n* = 142). Both species richness and abundance decreased from May to August (Figure S5). Weather variables and photoperiod had a clear seasonal pattern throughout the study period: temperature ranged from 16.5°C to 29.06°C (24.35 ± 2.46, mean ± SD), rainfall from 0 to 17.06 mm (3.89 ± 4.64), relative humidity from 34.42% to 85.75% (68.06 ± 11.89), and photoperiod from 631.8 to 793.2 min (720.6 ± 52.67; Figure S6).

The sample completeness curve stabilized around 250 individuals (Figure S7). The observed richness stabilized around 2150 individuals (Figure S8), while the higher orders of the diversity profile which give different weights to rare (*q* = 1) and abundant species (*q* = 2) stabilized around 250 individuals. The extrapolated component of the effective number of species (*q* = 0) estimated only one additional species with increasing individuals sampled. These results demonstrate that our sampling effort was sufficient to estimate species diversity (Figure S8).

**FIGURE 2:**
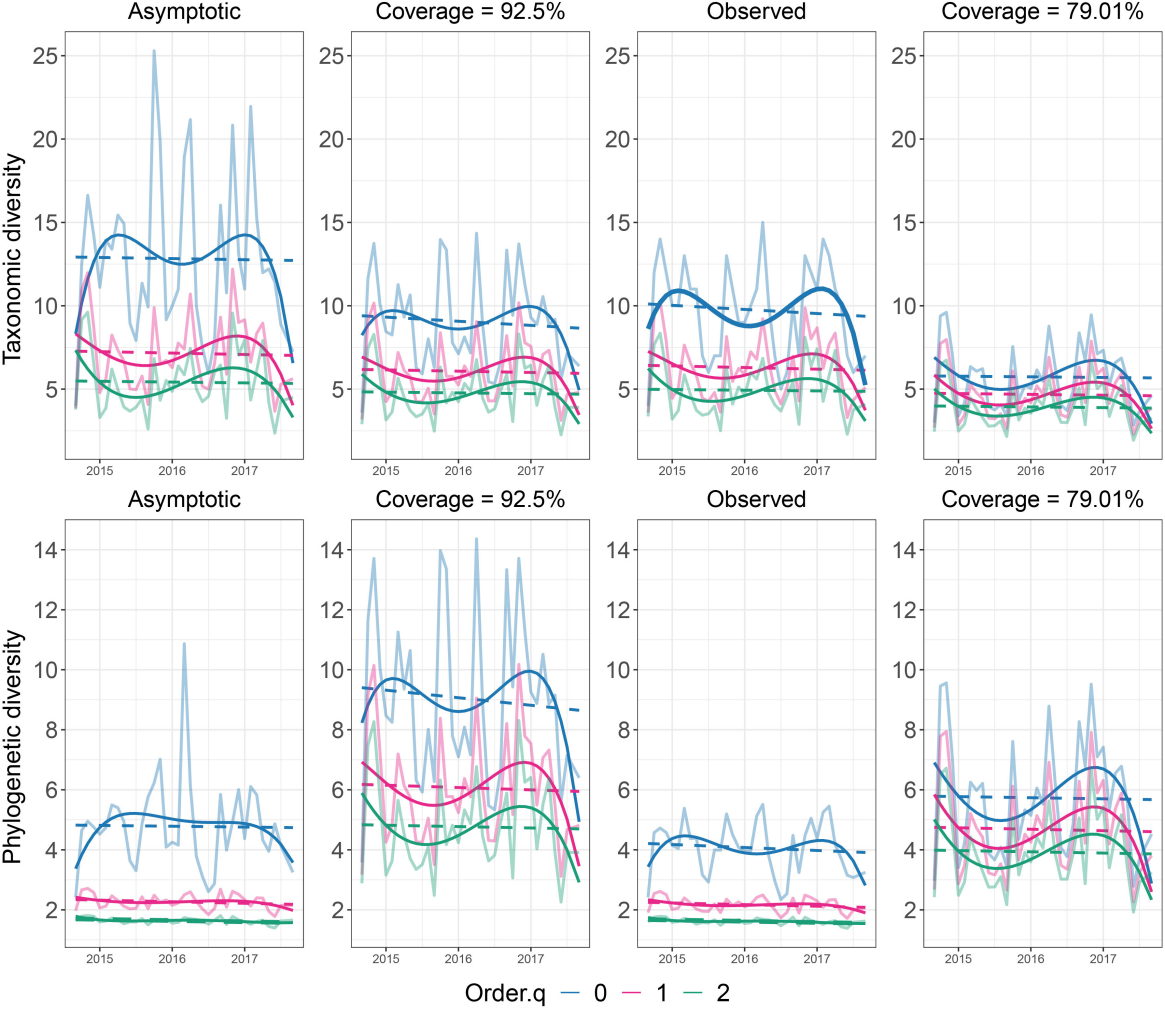
Temporal alpha diversity patterns for taxonomic (TD) and phylogenetic diversity (PD) built with frog species abundance data sampled each month throughout three consecutive years (2014–2015, 2015–2016, 2016–2017); *q* = 0 (blue), *q* = 1 (pink), and *q* = 2 (green) represent Hill numbers. Columns represent respectively, curves for asymptotic estimates, standardized estimates under a coverage value of confidence interval max = 92.5%, observed diversity, and standardized estimates under a coverage of confidence interval min = 79.01%. All curves were fitted by a quartic polynomial (continuous line) and a linear trend (dashed line). Statistical significance is represented by a bold curve.

Both taxonomic (TD) and phylogenetic alpha diversity (PD) varied little among years (Figure 2), but more pronouncedly within years. The observed TD was 10 species, while the expected TD was around 14 species (Figure 2). Only *q* = 0 of TD changed significantly over time, whereas rare (*q* = 1) and abundant TD (*q* = 2) changed little. There were no significant changes throughout years for phylogenetic alpha diversity (Figure 2). At least four lineages were observed (*q* = 0), but five were estimated. Both observed PD for rare (*q* = 1) and abundant species (*q* = 2) had a low variation, with about two lineages over years.

**FIGURE 3:**
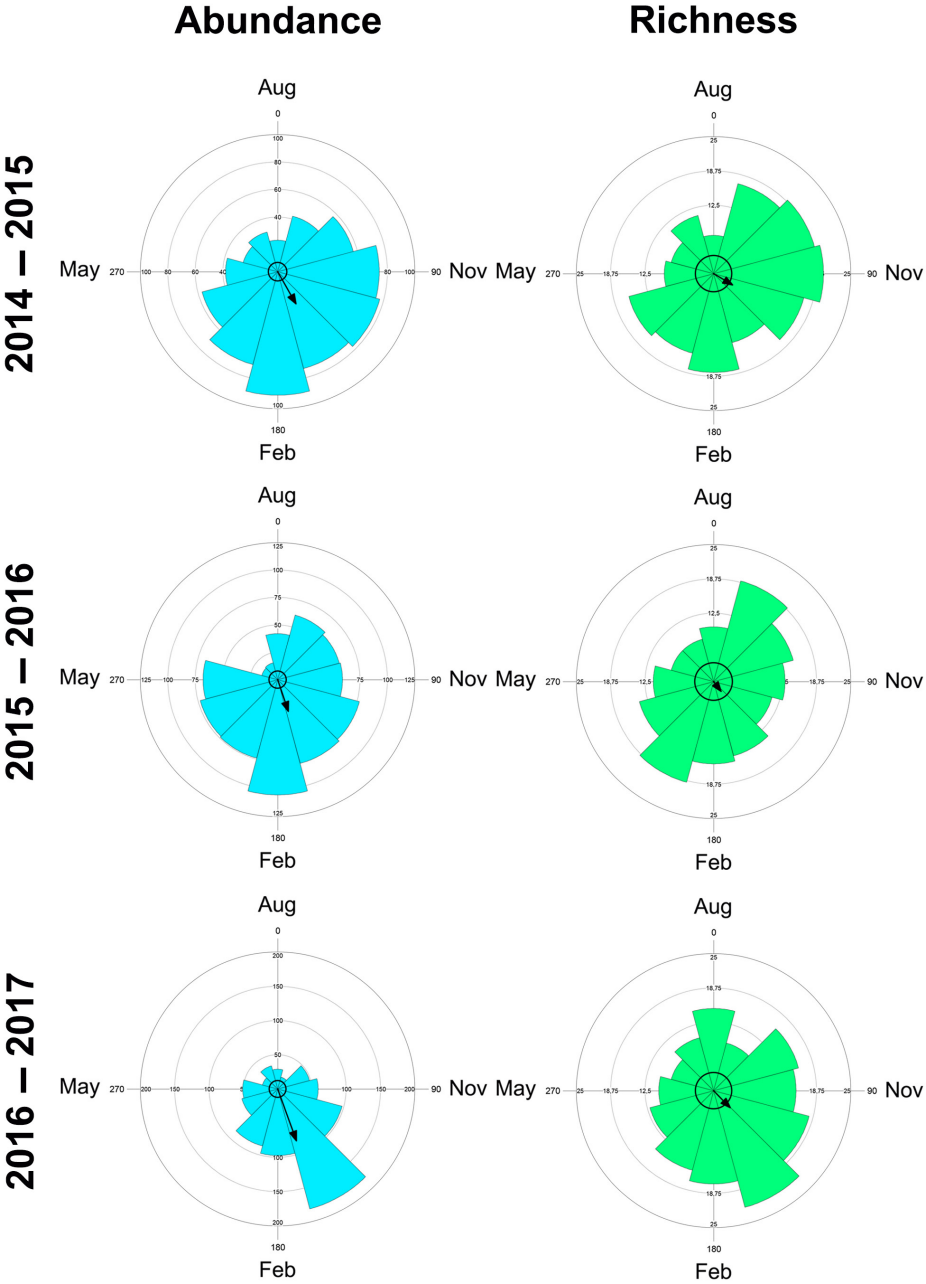
Rose diagrams showing the results of circular analyses for abundance (light blue) and species richness (light green) recorded from August 2014 to August 2017. Angles are equivalent to months. The mean vector length (*r*) is a measure of concentration of data around the year. The larger the vector, the higher the data concentration.

Species richness and abundance had a clear seasonal pattern in all years, except for richness in 2016 (Figure 3). Species richness was concentrated in December from 2014 to 2015 and from 2016 to 2017 (Table 1), while abundance was concentrated in January (Table 1; Figure 3).

**TABLE 1:** Results of circular statistical analysis testing seasonality in species richness and abundance.

Year	2014–2015		2015–2016		2016–2017	
Variable	Abundance	Richness	Abundance	Richness	Abundance	Richness
Observations (*n*)	657	170	727	157	778	167
Mean Vector (*α*)	150.347°	120.9°	161.279°	142.911°	160.151°	135.653°
Length of mean vector (*r*)	0.27	0.16	0.243	0.089	0.401	0.172
Circular Standard Deviation (SD)	92.757°	109.623°	96.375°	125.911°	77.474°	107.434°
Rayleigh's *Z*	47.79	4.372	42.931	1.255	125.006	4.963
*p*	**< 0.01**	**0.013**	**< 0.01**	0.285	**< 0.01**	**0.007**

*Note:* Significant *p* values in bold.

**FIGURE 4:**
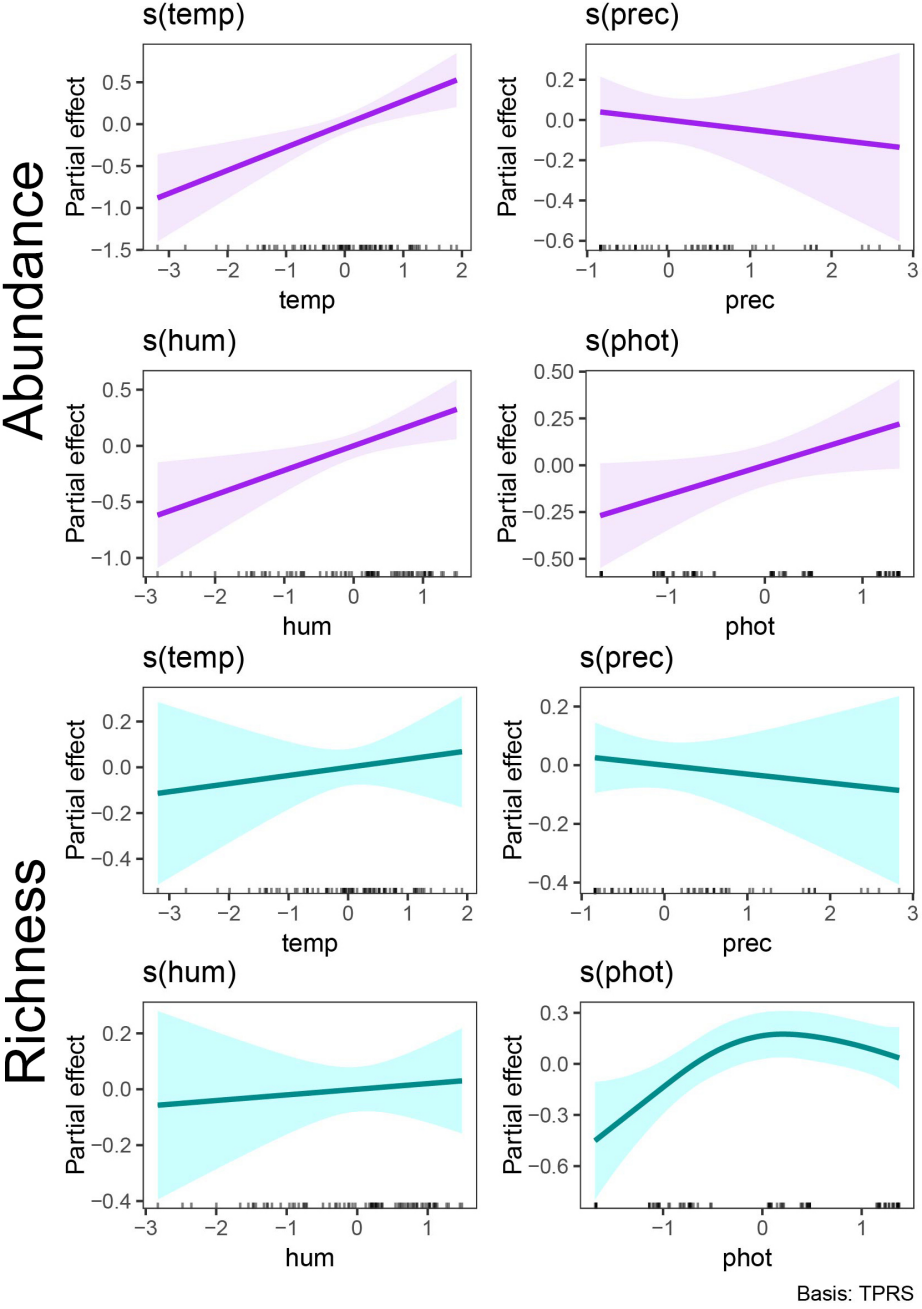
Results of Generalized Additive Mixed‐Effects Models (GAMM) for species abundance and richness, showing partial effect of each predictor variable and their relationships with abundance and richness, separately. Lines represent smoothing, and shadows represent confidence interval.

Abundance was positively related to humidity, temperature, and photoperiod, and negatively to rainfall, but this relationship was not significant (see Figures 4 and S9; Table 2). Species richness was positively related to photoperiod, and weakly to temperature and relative humidity, and negative to rainfall, but these relationships were not significant (see Figures 4 and S9; Table 2). Interestingly, the relationship between richness and photoperiod was non‐linear, since it peaked at intermediate values of photoperiod (Figure 4).

**TABLE 2:** Results of Generalized Additive Mixed‐Effect Models (GAMM) for species abundance and richness.

Variables	Abundance				Richness			
	edf	Ref.df	*F*	*p*	edf	Ref.df	*F*	*p*
s(temp)	1.000	1.000	11.575	**0.00108**	1.000	1.000	0.331	0.5670
s(prec)	1.000	1.000	0.339	0.56246	1.000	1.000	0.296	0.5878
s(hum)	1.000	1.000	6.995	**0.00998**	1.000	1.000	0.118	0.7325
s(phot)	1.000	1.000	4.214	**0.04362**	2.255	2.255	4.501	**0.0127**

*Note:* Significant *p* values in bold. *R* ^2^_adj_ = 0.106 for abundance and *R* ^2^_adj_ = 0.120 for richness.

Abbreviations: (hum), relative humidity; (phot), photoperiod; (prec), precipitation; (temp), temperature; edf, effective degrees of freedom (edf = 1 is equivalent to a linear relationship; edf > 1 and ≤ 2 is a weakly non‐linear relationship; edf > 2 highly non‐ linear relationship); *F*, Fisher's statistic; Ref.df, reference degrees of freedom; S, smooth terms.

**FIGURE 5:**
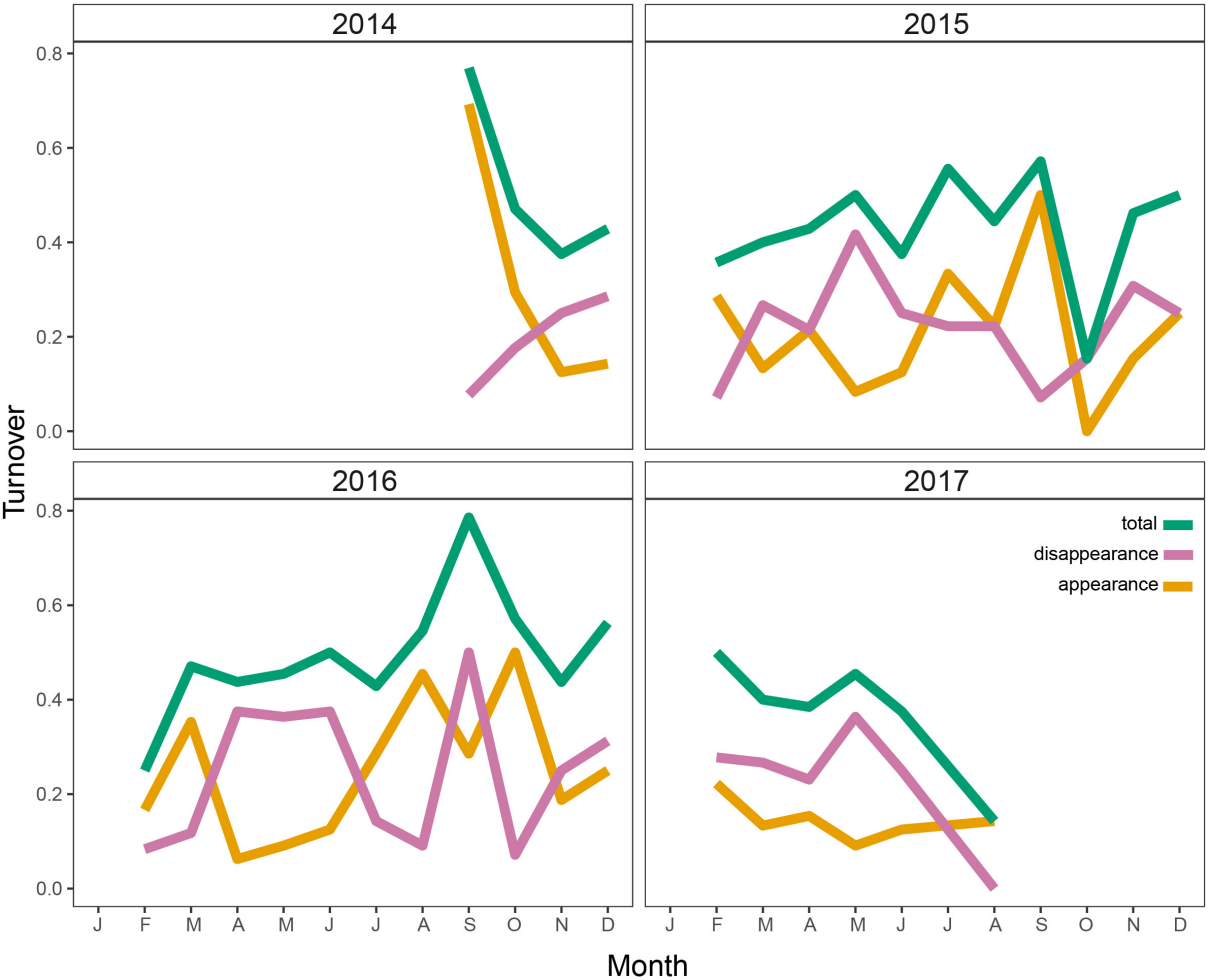
Species turnover within and among years showing total turnover, disappearance, and appearance.

**FIGURE 6:**
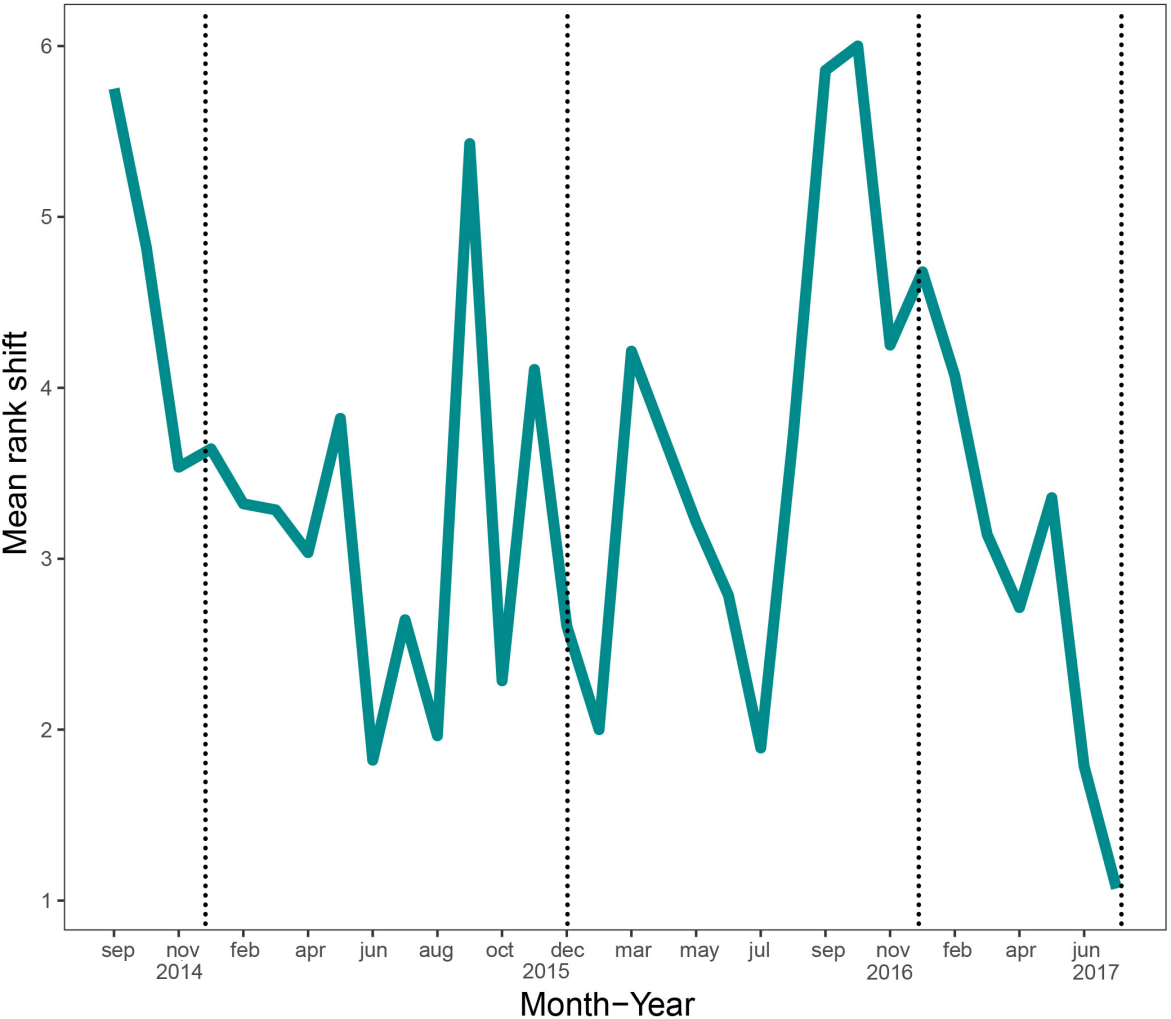
Mean abundance Rank Shift per month throughout 2014, 2015, 2016, and 2017. Dotted lines denote the end of each year.

Total beta diversity, disappearance, and appearance were highest within years, with disappearance increasing mainly in colder months, while appearance increased in hotter months (i.e., October through March; Figure 5). Also, the total turnover was greatest in 2015 and 2016, especially between June and November (transition dry‐rainy season), due to the balance between species disappearance and appearance (Figure 5). Months with higher than average (MRS 3.45) abundance rank shifts were September 2014 (MRS 5.75), October 2014 (MRS 4.82), September 2015 (MRS 5.42), March 2016 (MRS 4.21), September 2016 (MRS 5.85), October 2016 (MRS 6.00), and December 2016 (MRS 4.67; Figure 6). Months with lower‐than‐average shifts were June 2015 (MRS 1.82), August 2015 (MRS 1.96), July 2016 (MRS 1.89), June 2017 (MRS 1.78), and August 2017 (MRS 1.07; Figure 6).

**FIGURE 7:**
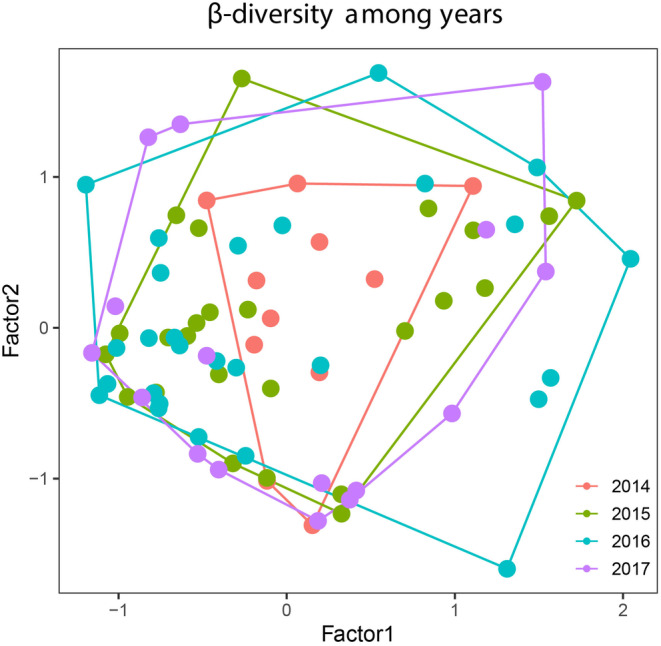
Ordination diagram showing the result of the model‐based unconstrained ordination showing temporal beta diversity among years.

Species composition differed among years (Wald χ^2^ = 11.11; df = 3, 75; *p* = 0.001), mostly between 2014 and subsequent years and between 2015 and 2017 (Table 3). The differences between 2014 and subsequent years and 2015 and 2017 can be related to the beginning and ending of the survey. Also, our results showed little compositional variation among years, notice the centroids were close to each other and convex hulls for each year were nested in the ordination diagram (Figure 7). This can be due to the same species occurring over the 3 consecutive years (e.g., *L. podicipinus, D. minutus*, *D. nanus* , and *B. punctata* ; see Figure S10). Species that contributed to compositional variation among years were *Rhinella diptycha* (*p* = 0.022) and *Leptodactylus fuscus* (*p* = 0.075).

**TABLE 3:** Results of the post hoc test of the model‐based approach for differences in species composition among years.

	Year 2014	Year 2015	Year 2016	Year 2017
2014	—	74.03	59.46	68.93
2015	**0.005**	—	41.68	62.51
2016	**0.010**	0.120	—	17.95
2017	**0.005**	**0.010**	0.835	—

*Note:* Lower triangle shows *p* values, and upper triangle shows observed statistics (pairwise distances between years). Significant *p* values in bold.

## 4 Discussion

Our results showed that both taxonomic and phylogenetic diversity varied more within than between years. This concurs with the fact that species abundance and richness were highly seasonal and influenced mainly by humidity, temperature, and photoperiod. Additionally, species composition varied more within than between years, driven specially by species turnover at the transition between colder and hotter months. Abundance rank shift varied more within years, mainly due to shifts in the occurrence of dominant species. Therefore, recurrent ecological time (seasonality) seems to be more important than cosmological time (variation among years) for determining the temporal dynamics of this frog community.

Taxonomic alpha diversity varied more within than between years. The low temporal alpha diversity could be explained by few ecological opportunities for priority effects between years (Fukami 2015; Leibold and Chase 2018). This is because the most abundant species (e.g., *Leptodactylus podicipinus* and *Dendropsophus minutus* ) occurred throughout all years. This mean that dominant species are using a large proportion of the available resources (Fukami 2015), decreasing the chance of colonization by other species and consequently decreasing taxonomic diversity through time. Furthermore, less productive regions, like the Brazilian Cerrado in which we conducted this study, are more temporarily predictable (Chase 2010), filtering out species less adapted to drought, which decrease species diversity when compared to more productive regions (Gomes‐Mello et al. 2021). Additionally, alpha diversity seems to respond to seasonal changes, since some species in our study were cold tolerant (i.e., *L. podicipinus* and *D. minutus* ), allowing them to occur throughout the year, while other species appeared in hotter and disappeared in colder seasons. Therefore, the low temporal alpha diversity was influenced mainly by seasonal changes, selecting cold tolerant species and those with high population sizes.

Phylogenetic alpha diversity did not vary over years. This was the first study that evaluated temporal phylogenetic alpha diversity of frogs over 3 consecutive years in a peri‐urban area in a tropical country. This result might be explained by the small phylogenetic scale of the community, with only two lineages (i.e., Hylidae and Leptodactylidae) dominating the community through time, while others (i.e., Phyllomedusidae and Bufonidae) were represented by only one species each. Ecological processes depend on both phylogenetic (Graham, Storch, and Machac 2018) and spatial scales (Levin 1992; Leibold and Chase 2018). The smaller the scale of the study, both in terms of grain and extent (as is our case), the smaller the environmental heterogeneity encompassed, making turnover less likely. Moreover, Neotropical anuran communities phylogenetically clustered in less productive environments, where available energy is also low, making calling activation energy low and allowing species to be active even in colder months (Canavero et al. 2019). Our results agree with this, provided that temperature did not influence species richness and the community was composed of a few clades only. Additionally, in harsh and less productive environments (e.g., *Cerrado* domain, Brazil), the regional species pool is impoverished compared to forested regions, both in terms of the total number of species and lineages present (e.g., Benício et al. 2021). This is because only tolerant species can occur in those regions, due to low evolutionary rates of physiological traits that confer thermal tolerance and decrease evaporative water loss (Bennett et al. 2021; Pottier et al. 2022; Sasaki et al. 2022). Likewise, a small phylogenetic extent might also constrain the variation through time of the diversity metrics calculated (see Graham, Storch, and Machac 2018), which might explain the low variation in all *q* orders of PD we found, especially because they capture structure toward the tip level (Chao et al. 2021). Therefore, the seasonal variation in weather variables in the study site, although considerably high, is not sufficient to change PD.

Species abundance and richness had a clear seasonal pattern that was positively correlated with temperature, relative humidity, and photoperiod for abundance and photoperiod for richness. Breeding activity in anurans is commonly concentrated in the wet season in tropical environments (e.g., Aichinger 1987; Canavero and Arim 2009; Ceron et al. 2020), mainly due to the physiological dependence of frogs to rainfall and warm temperatures (Duellman and Trueb 1994; Wells 2007). Most frog species in the Cerrado breed in temporary ponds (Valdujo et al. 2012, 2013; Vasconcelos et al. 2019). Therefore, temperature and rainfall levels seem to be good environmental cue pointing to favorable conditions for reproduction (Aichinger 1987; Kopp and Eterovick 2006), since they can indicate that ponds are filled with enough water and desiccation risk is decreased, providing favorable conditions for tadpole survival (Jared et al. 2020). Surprisingly, rainfall affected neither abundance nor species richness. Additionally, likely due to low thermal tolerance, warm‐adapted species in our study (e.g., *D. nanus* , *B. raniceps* , *B. punctata* ) decrease their occurrence and breeding in cold dry months. Photoperiod was the most important variable determining temporal distribution of species richness. It agrees with other studies from temperate and tropical areas in which photoperiod is an important environment cue used by frogs to predict favorable conditions (e.g., Both et al. 2008; Canavero and Arim 2009; Fuentes‐de la Rosa, Ochoa‐Ochoa, and Canavero 2021). However, when days become long, there was a decrease in richness in our study, suggesting a non‐linear association between richness and photoperiod. Abundance was seasonal and concentrated on the same months every year. This is because these months were the hottest and rainiest. Furthermore, it could indicate that reproductive phenology exhibits a stasis at this temporal scale. A stasis or the absence of phenological shift happens when there are no drastic changes in weather variables, allowing species to maintain natural reproductive cycles due to environmental predictability (Post 2019). In conclusion, photoperiod was the main cue determining species richness; while temperature, humidity, and photoperiod drove abundance. Therefore, these were the best cues allowing frogs to track environmental conditions in our study site, while precipitation seems not so crucial.

Abundance rank shift had a remarkable variation within years but little change between years. This suggests that there was a shift in species dominance pattern across seasons. These shifts in relative species abundance can promote species coexistence, because they allow for seasonal resource (e.g., microhabitat use and food) partitioning (Tokeshi 1999). However, the abundance of some species (e.g., *L. podicipinus* and *D. minutus* ) was continuously high throughout years. In this case, species might monopolize resources (Tokeshi 1999), reducing their availability for later colonizers. Therefore, there are few opportunities for new species to establish in the environment, decreasing the chance of rank shift between years. Additionally, the most abundant species occurred in all years across all seasons, while other species disappeared in colder months (e.g., *D. nanus* and *B. punctata* ). Thus, different life‐history strategies in the community might have contributed to abundance rank shifts within a year, with continuous breeders representing core species, while those with occasional occurrence were satellite species (Hanski 1982). Abundance rank shifts might also be a result of seasonal fluctuations in resource availability across seasons. Frogs mainly feed upon arthropods (see Wells 2007). Recent studies (e.g., Ceron et al. 2022) have found that differences in resource availability through time are the mechanism responsible for interaction rewiring in frog‐arthropod food webs. The resource‐ratio hypothesis postulates that species that maintain a zero net growth rate using less resources can outcompete other species (Tilman 1982). Therefore, species that require more resources to maintain their population at equilibrium probably will be excluded if resources fluctuate in time. In conclusion, abundance rank shifts within years were driven mainly by the relationship between dominant and satellite species.

Species composition varied more within than between years, mainly driven by seasonal variation in weather variables. Differences in compositional variation between the first and subsequent years and between the second and last year might be explained by a sampling effect, probably due to beginning and end of the sampling. The ordination diagram suggests that temporal beta diversity seems to be due to nestedness rather than turnover. The temporal pattern of nestedness in amphibian communities is associated with physiological constraints due to temperature seasonality. As a result, species tend to co‐occur in time more than expected by chance, increasing modularity, which is positively associated with community stability (Canavero, Arim, and Brazeiro 2009; Canavero et al. 2019). Our results support that statement, given that species composition was relatively stable throughout the study period. However, Ganci et al. (2022) evaluating spatial beta diversity along a rural–urban gradient in the same city found that beta diversity was driven by turnover. This suggests that the strength of environmental filters determining temporal species composition (Leibold and Chase 2018) depends on where the community is along the urbanization gradient. The low *R* ^2^ of our model might also suggest that neutral stochasticity plays a relevant role in determining species distribution through time. Stochastic events might promote changes in community dynamics as a response to random change in demography or the environment (Vellend et al. 2014). Likewise, urbanized sites are harsh environments (Lourenço‐de‐Moraes et al. 2018; Yang, Zhao, and Liu 2022), and communities inhabiting these sites might be less prone to stochasticity (see Chase 2010) with species composition fluctuating less through time. Therefore, compositional differences within a year might be related to species turnover among seasons, while low variation in species composition between years might be related to neutral stochasticity in a harsh and less productive environment.

The temporal variation in selection is weaker inter‐ than intra‐annually because recurrent ecological time is more limited in seasonal environments than cosmological time (Post 2019). The constraints imposed by physiological responses of amphibian species to weather variables (Feder and Burggren 1992; Hillman et al. 2008; Canavero et al. 2018) are a stronger determinant of species occurrence, limiting the recurrent ecological time available to species exhibit phenophases, than the cosmological passage of time. Although a previous meta‐analysis pointed out that the strength and direction of selection vary among years (Siepielski, DiBattista, and Carlson 2009), it seems that traits relevant for ecological success in this community are not under strong selection, or there might be some kind of cryptic eco‐evolutionary dynamics (Kinnison, Hairston Jr., and Hendry 2015). Some papers have suggested that urban environments can even foster cryptic eco‐evolutionary dynamics (Carroll 2008; Brans et al. 2017; Hendry, Gotanda, and Svensson 2017). While we did not investigate these aspects in this study, they should eventually be subject to further research.

In conclusion, our peri‐urban community changed little in a mid‐timescale, which might to be a response to a small variation in weather variables across years. This suggests that peri‐urban areas with temporally stable weather conditions might still hold a considerable diversity. However, our study evaluated a single site through time. So far, no study evaluated how weather variables affects species diversity along rural–urban gradients at mid to long timescales (see Severgnini et al. 2024). Thus, more studies are necessary to understand how communities change through time and how weather variables affects community dynamics. This can help answering several questions in ecology, especially how species adapt and survive in an increasingly urbanized planet under climate change.

## Author Contributions

**Marcos R. Severgnini:** data curation (lead), formal analysis (lead), methodology (lead), visualization (lead), writing – original draft (lead). **Mônica M. de Oliveira:** data curation (supporting). **Luciana M. Valério:** project administration (equal), resources (equal), supervision (supporting). **Diogo B. Provete:** conceptualization (lead), formal analysis (equal), methodology (equal), supervision (lead), writing – review and editing (lead).

## Ethics Statement

ICMBio provided collecting permit (#50590).

## Conflicts of Interest

D.B.P. is Senior Editor of Ecology and Evolution.

## Supporting information


Data S1.

## Data Availability

All data and associated R code used to run the analysis are available at FigShare https://doi.org/10.6084/m9.figshare.24470161.v2.
