# Supplementary material for: Temporal Dynamics of Species Richness and Composition in a Peri‐Urban Tropical Frog Community in Central Brazil
Source: Ecol Evol. 2024 Nov 25;14(11):e70628. doi: 10.1002/ece3.70628 (PMC11588356; doi:10.1002/ece3.70628)
Supplement: Supplementary file 1 — Data S1. [file ECE3-14-e70628-s001.pdf]

Temporal dynamics of species richness and composition in a peri-urban tropical frog community in central Brazil

Marcos R. Severgnini[1,2*], Mônica M. de Oliveira[2,3], Luciana M. Valério[2,4], Diogo B. Provete[3,5,6]

[1] Graduate program in Ecology and Conservation, Institute of Biosciences, Federal University of Mato Grosso do Sul, Campo Grande, 79002970, Mato Grosso do Sul, Brazil

[2] Fragment Ecology Study Group, Campo Grande, 79032-290, Mato Grosso do Sul, Brazil

[3] Institute of Biosciences, Federal University of Mato Grosso do Sul, Campo Grande, 79002970, Mato Grosso do Sul, Brazil

[4] Catholic University Dom Bosco, Campo Grande, 79117-010, Mato Grosso do Sul, Brazil

[5] Gothenburg Global Biodiversity Centre, PO Box 100, Göteborg, Sweden.

[6] German Centre for Integrative Biodiversity Research -iDiv, Halle-Jena-Leipzig, Leipzig, Germany.

*Corresponding author *e-mail*: marcos.severgnini@ufms.br

Supplementary material

Tables, legends and figures

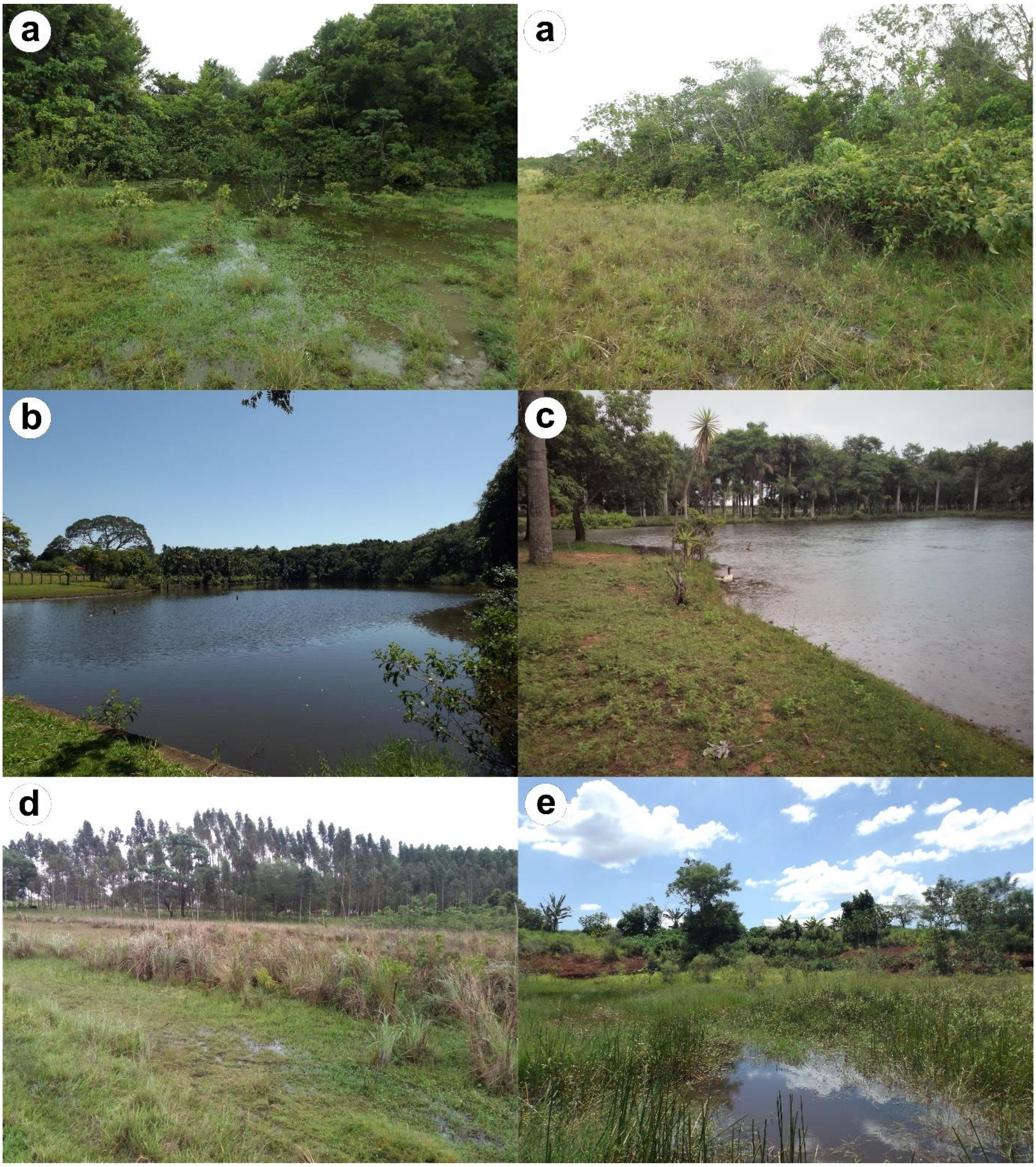

Figure S1. Temporary and permanent ponds surveyed for frogs at the São Vicente Institute, Mato Grosso do Sul, central Brazil: (**a**) Temporary Pond with wet soil trampled by cattle; (**b**) Man-made permanent pond; (**c**) Man-made permanent pond in open area; (**d**) Natural temporary pond; (**e**) Natural temporary pond. (Photos by Mônica M. de Oliveira).

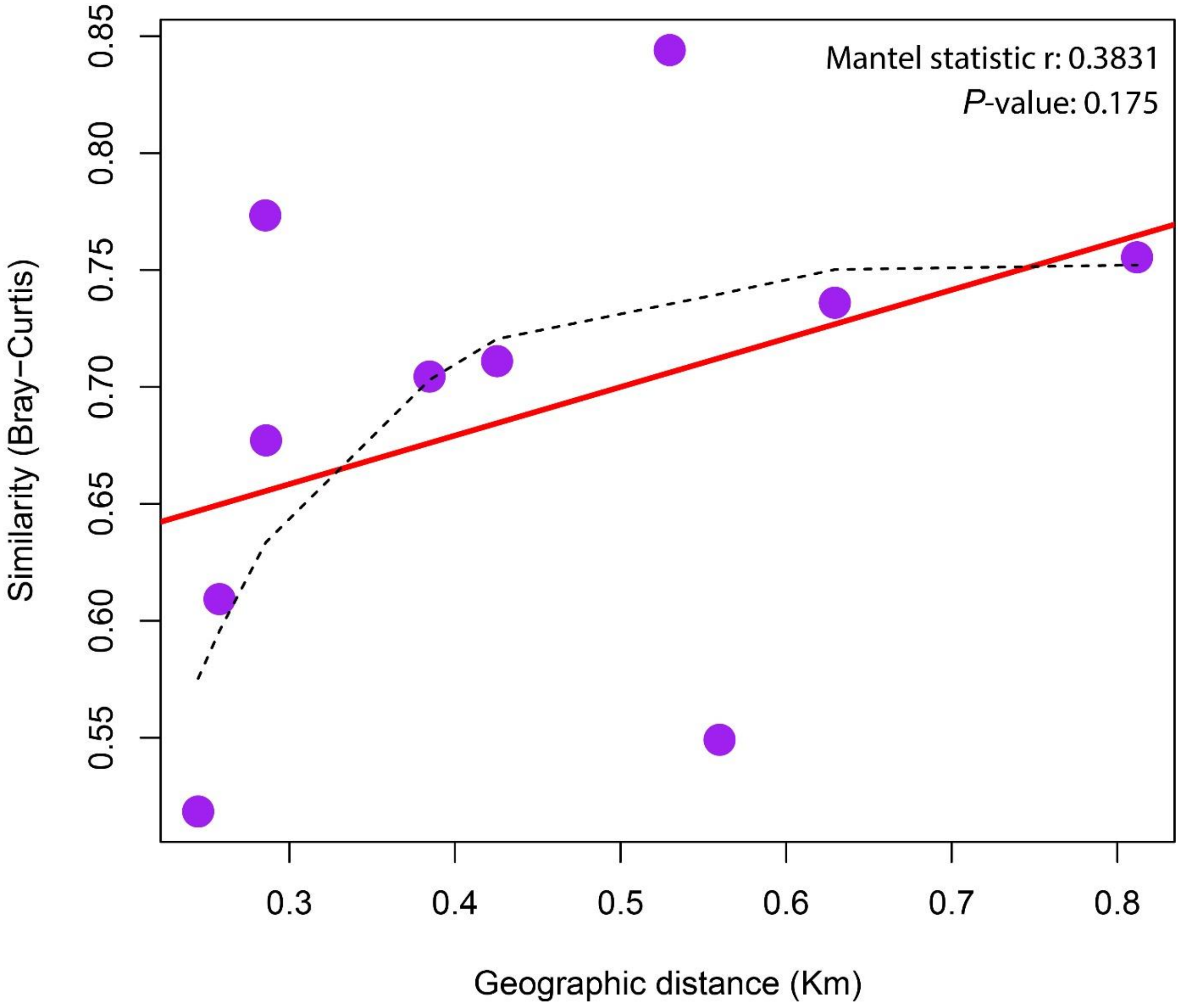

Figure S2. Relationship between composition similarity among ponds and geographic distance (Euclidean). Each purple point represents a pairwise distance between the five ponds sampled. The red line represents the positive relationship based on a Mantel correlation and the dashed line represent a smoothed curve. Notice that the smoothed line reaches a plateau at around 72% similarity, which means that ponds share about 72% of their species in average. There was no spatial autocorrelation in species composition either.

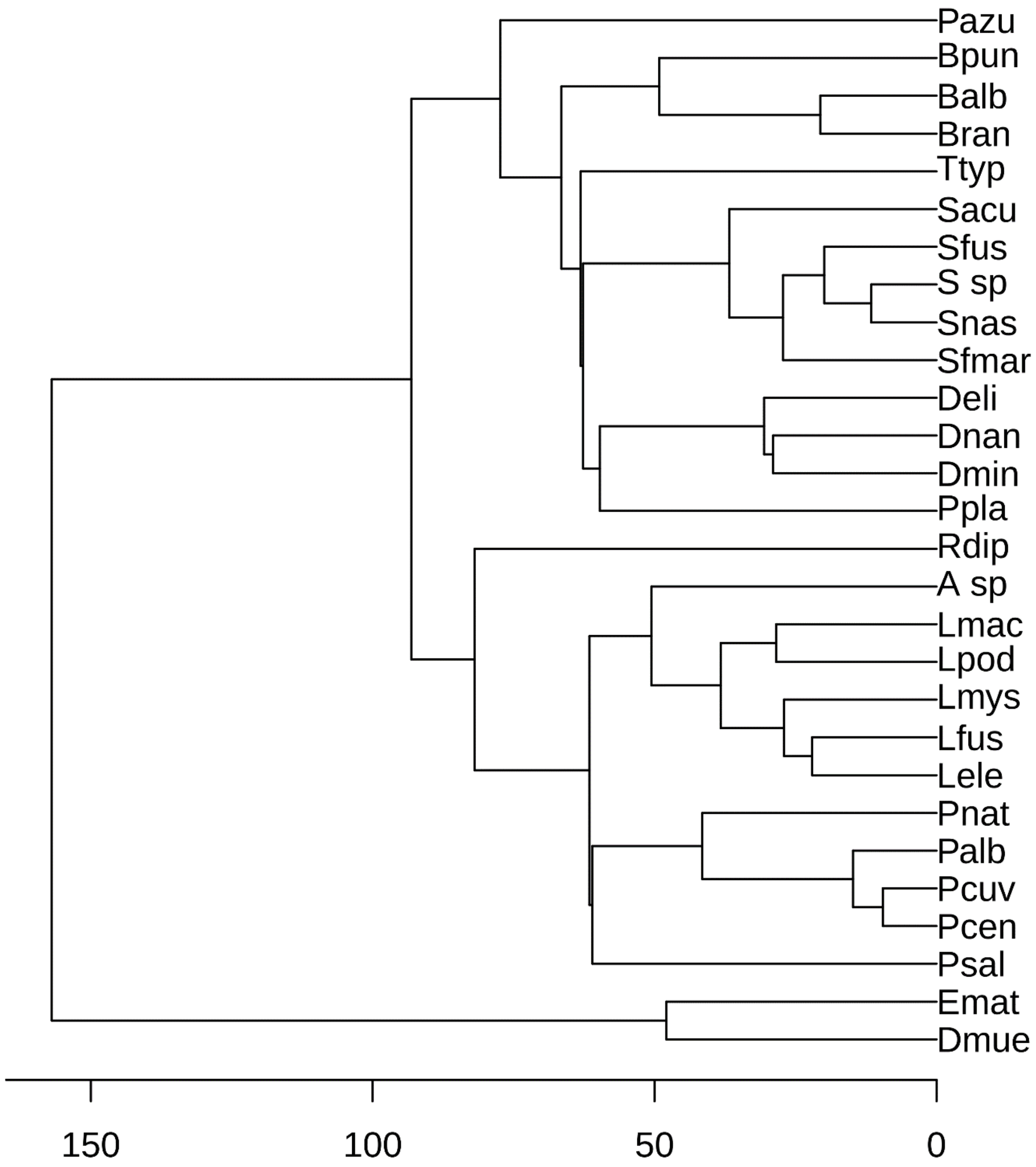

Figure S3. Phylogenetic tree obtained by pruning the fully-sampled, Maximum Clade Credibility Tree of Jetz and Pyron (2018) for 28 species found at the São Vicente Institute. For full species names, see Table S1.

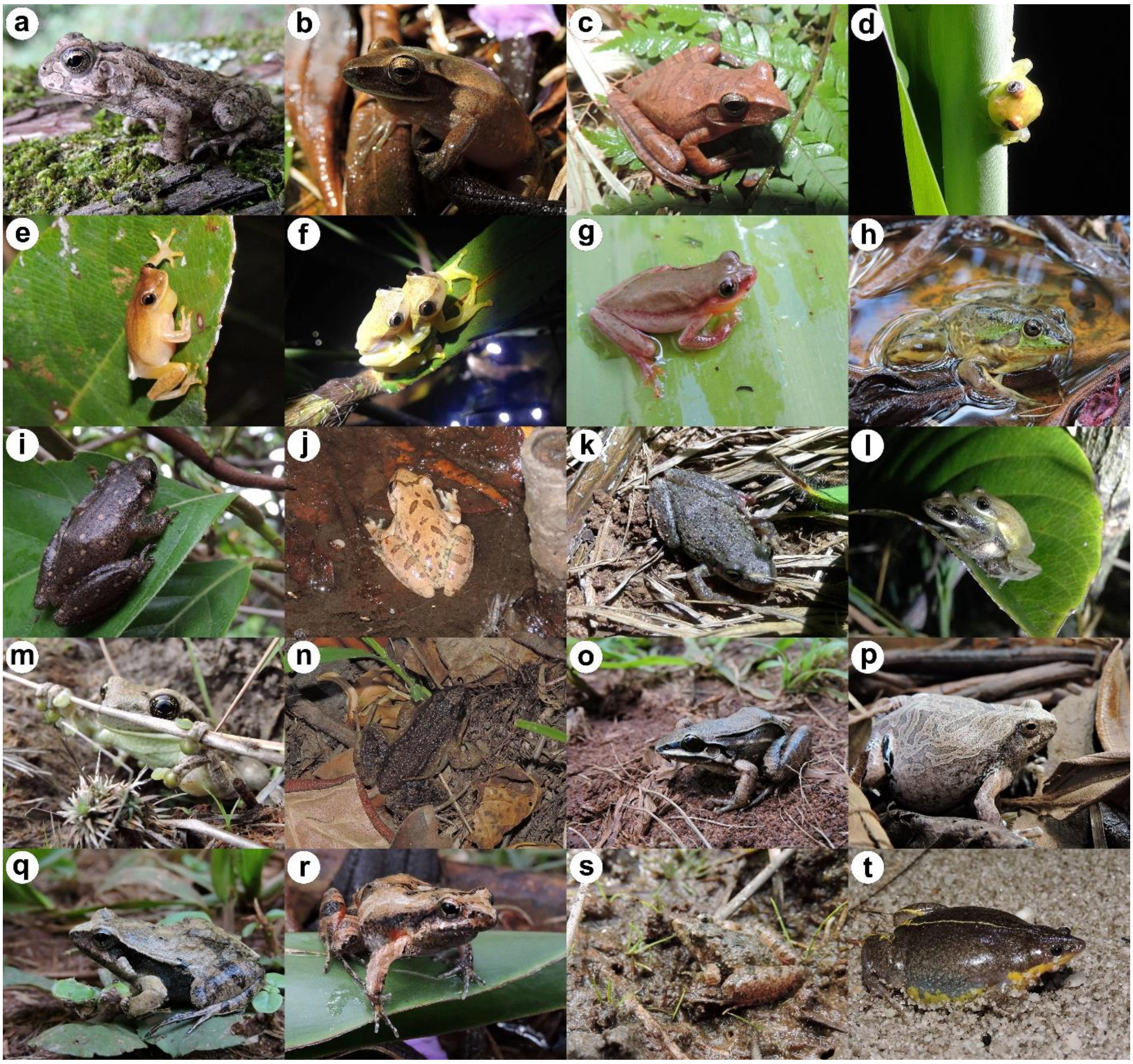

Figure S4. Frogs recorded at the São Vicente Institute, Campo Grande, Mato Grosso do Sul, central Brazil from August 2014 to August 2017: (**a**) *Rhinella diptycha*; (**b**) *Boana albopunctata*; (**c**) *Boana raniceps*; (**d**) *Boana punctata*; (**e**) *Dendropsophus minutus*; (**f**) *Dendropsophus nanus*; (**g**) *Dendropsophus elianeae*; (**h**) *Pseudis platensis*; (**i**) *Scinax* sp.; (**j**) *Scinax fuscovarius*; (**k**) *Scinax nasicus*; (**l**) *Scinax fuscomarginatus*; (**m**) *Trachycephalus typhonius*; (**n**) *Leptodactylus podicipinus*; (**o**) *Leptodactylus elenae*; (**p**) *Physalaemus nattereri*; (**q**) *Physalaemus albonotatus*; (**r**) *Physalaemus cuvieri*; (**s**) *Pseudopaludicola saltica*; (**t**) *Elachistocleis bicolor*. (Photos by: Allan C. Anjos, Mônica M. de Oliveira, and Marcos R. Severgnini).

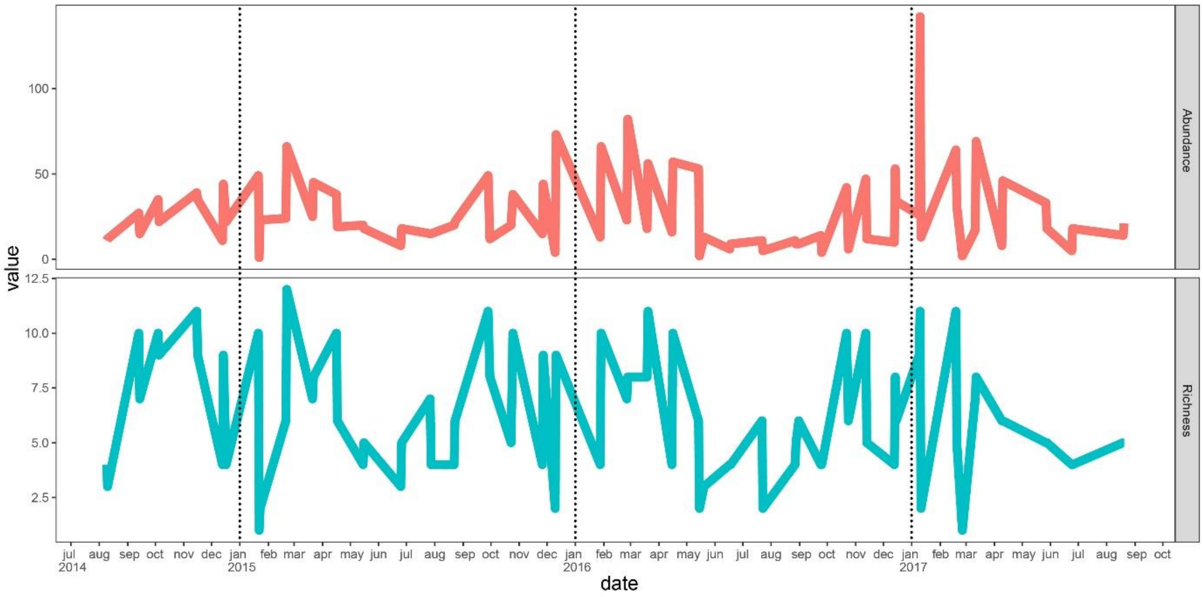

Figure S5. Frog abundance (above) and species richness (below) over the three years of sampling. Dotted lines denote the beginning of each year.

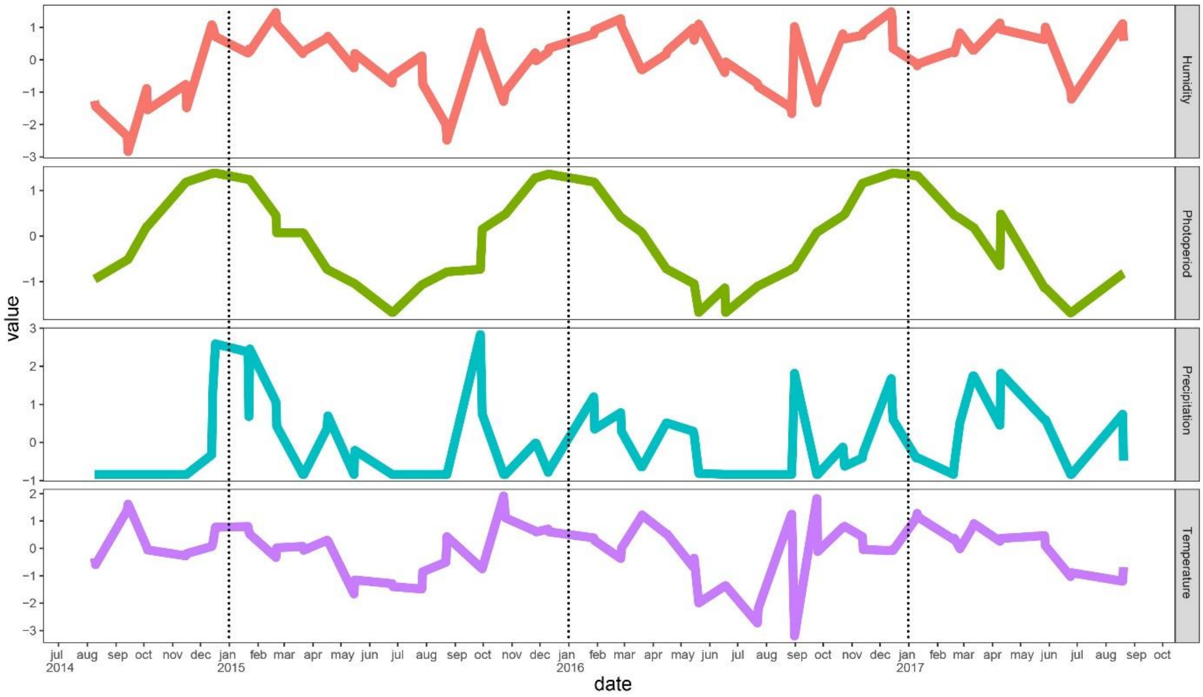

Figure S6. Climatic variables measured in the study site throughout the three years of sampling. Dotted lines represent the beginning of each year.

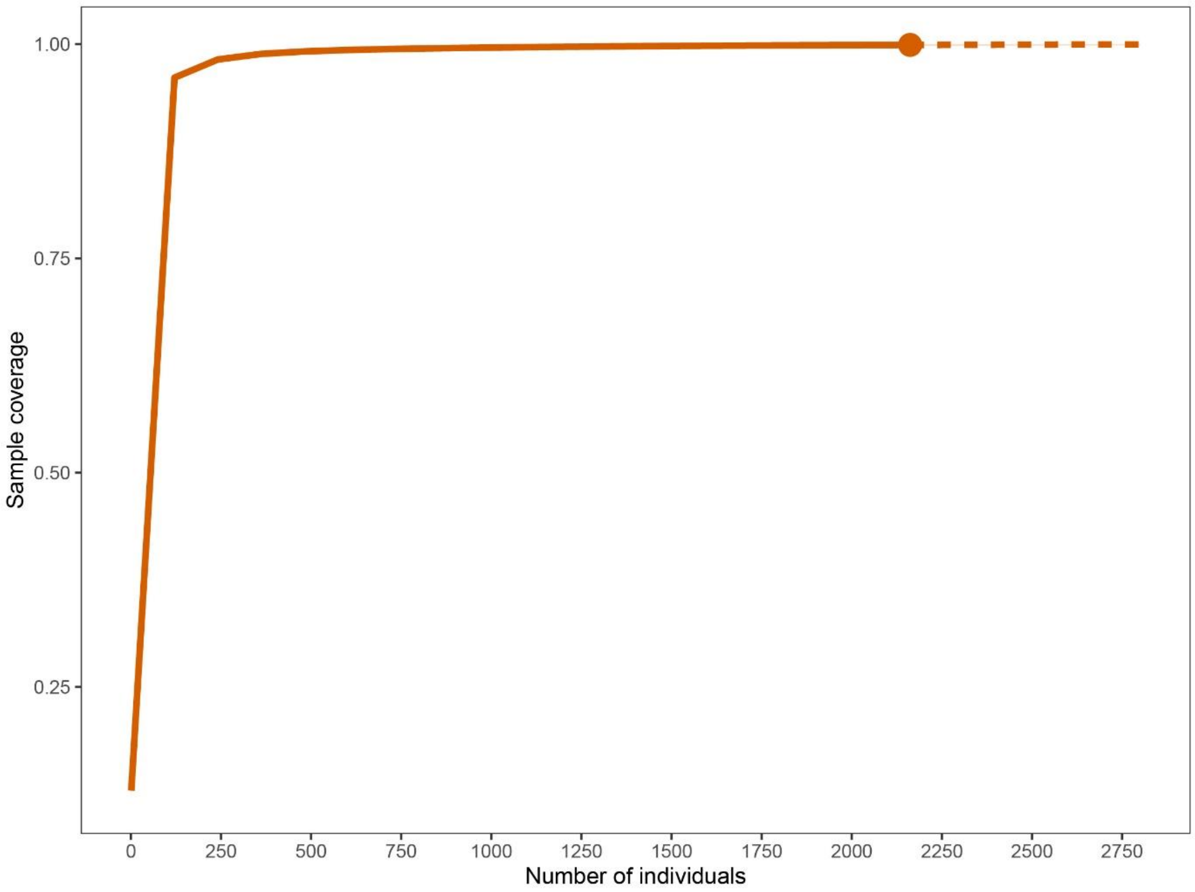

Figure S7. Sample completeness curve showing the number of individuals per sampling from August 2014 to August 2017. The continuous line represents observed number of individuals and dashed line represents estimated number of individuals.

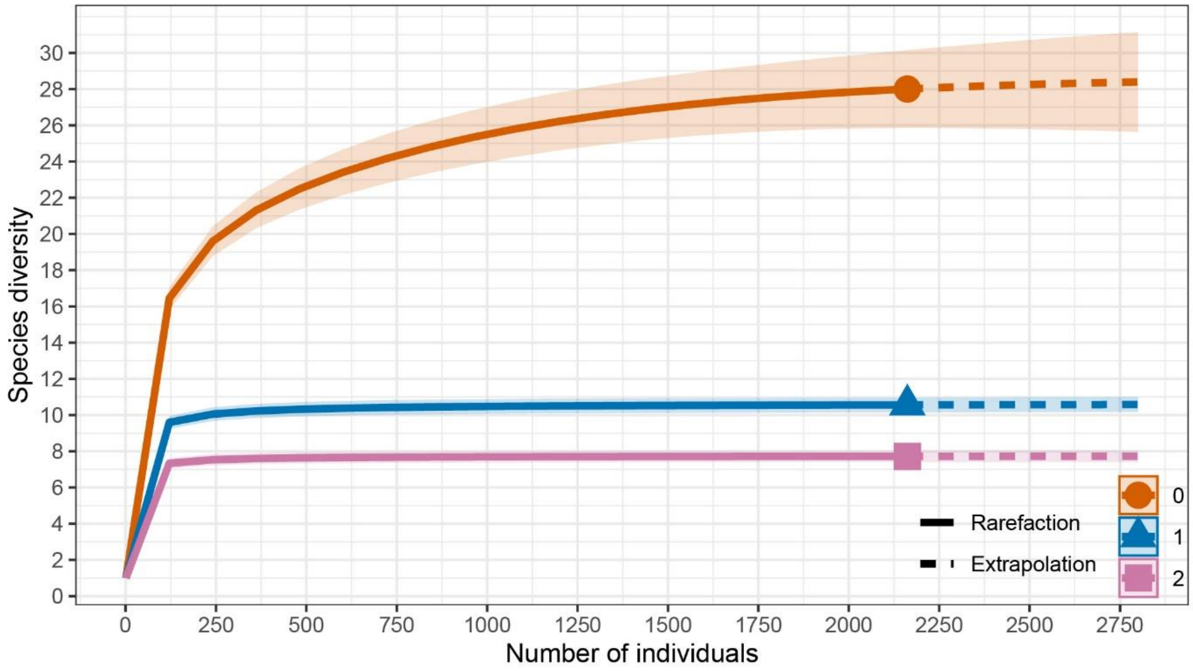

Figure S8. Individual-based rarefaction curve showing Hill numbers for frog species sampled from August 2014 to August 2017. The orange line represents the effective number of species (q = 0), blue line represents q = 1, which is equivalent to Shannon index, and pink line represents q = 2, which is equivalent to a Simpson index. Shades represent 95% confidence interval.

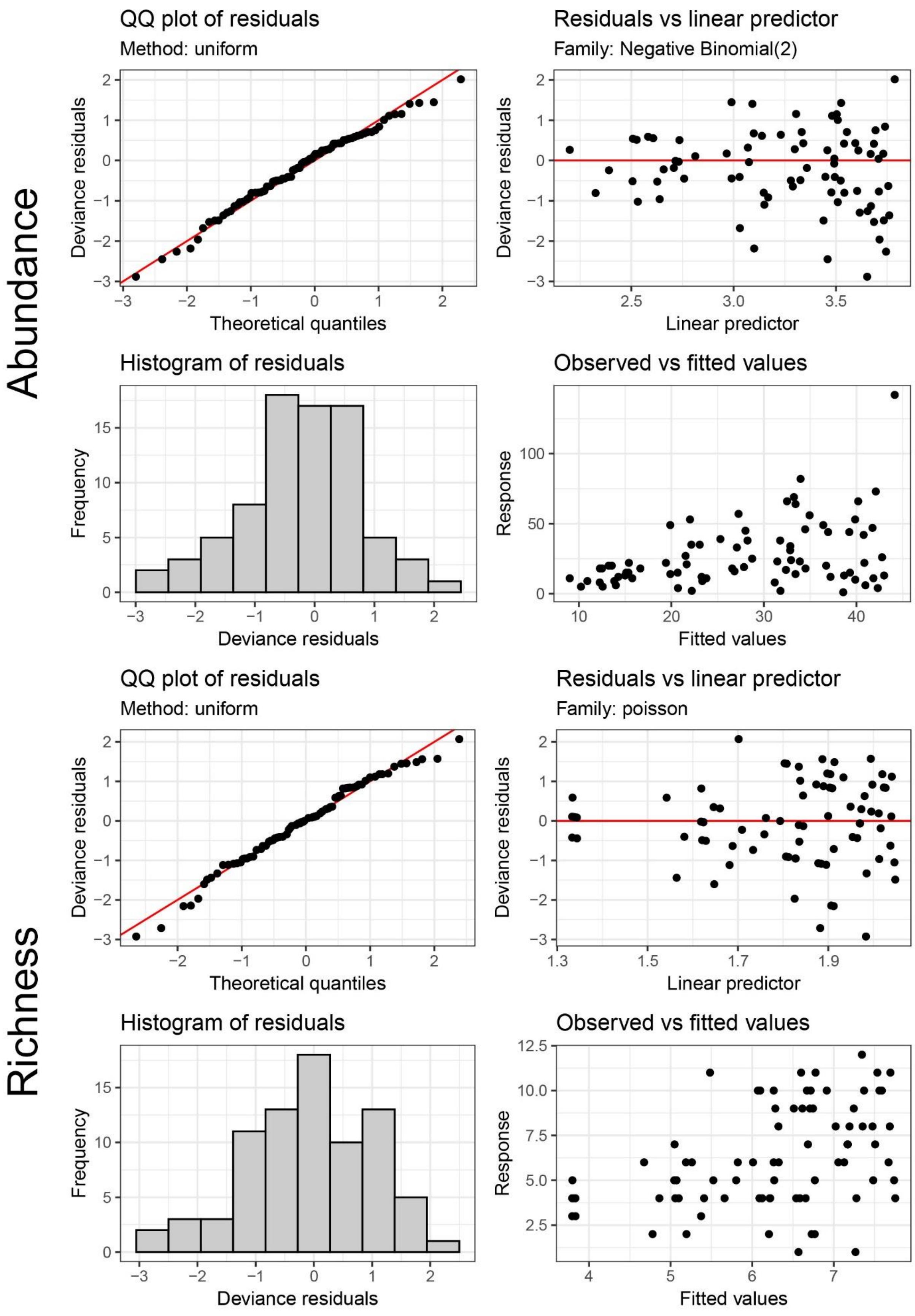
Abundance
QQ plot of residuals
Method: uniform
Deviance residuals
Theoretical quantiles
Residuals vs linear predictor
Family: Negative Binomial(2)
Deviance residuals
Linear predictor
Histogram of residuals
Frequency
Deviance residuals
Observed vs fitted values
Response
Fitted values
Richness
QQ plot of residuals
Method: uniform
Deviance residuals
Theoretical quantiles
Residuals vs linear predictor
Family: poisson
Deviance residuals
Linear predictor
Histogram of residuals
Frequency
Deviance residuals
Observed vs fitted values
Response
Fitted values

Figure S9. Residuals of Generalized Additive Mixed Models (GAMM) for abundance and species richness.

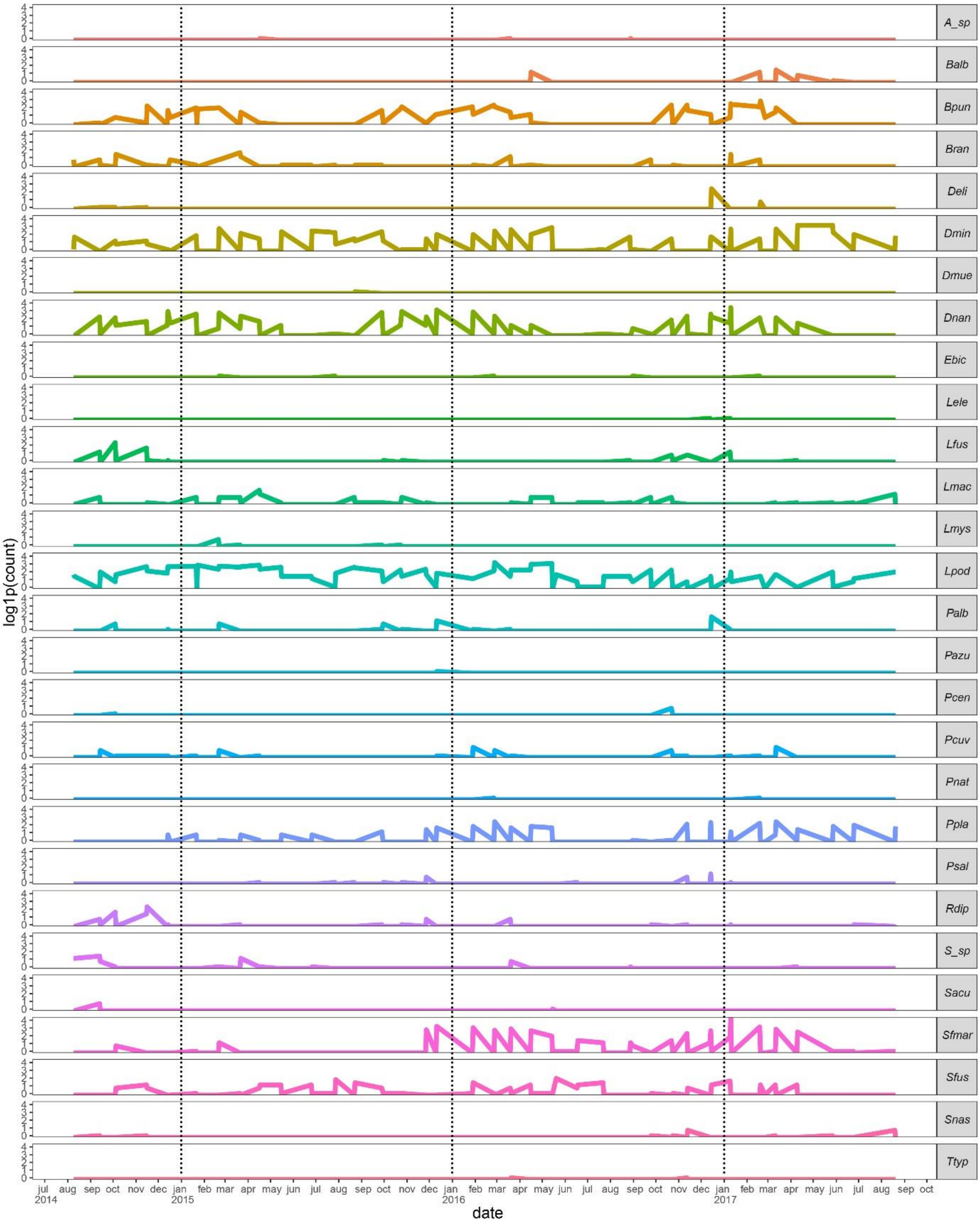

Figure S10. Abundance (log scale) of each species sampled over the three consecutive years of sampling (see Table S1 for full species names). Dotted lines denote the beginning of each year.

Table S1. Anurans recorded at the São Vicente Institute, Campo Grande, Mato Grosso do Sul, central Brazil from August 2014 to August 2017. Nomenclature follows Frost (2023).

**Taxa**	**Acronyms**	**Total Abundance**
**Bufonidae (1)**		
Rhinella diptycha (Cope, 1862)	Rdip	35
**Hylidae (13)**		
Boana albopunctata (Spix, 1824)	Balb	13
Boana punctata (Schneider, 1799)	Bpun	217
Boana raniceps (Cope, 1862)	Bran	47
Dendropsophus elianeae (Napoli & Caramaschi, 2000)	Deli	17
Dendropsophus minutus (Peters, 1872)	Dmin	294
Dendropsophus nanus (Boulenger, 1889)	Dnan	312
Pseudis platensis Gallardo, 1961	Ppla	127
Scinax acuminatus (Cope, 1862)	Sacu	3
Scinax fuscomarginatus (A. Lutz, 1925)	Sfmar	301
Scinax fuscovarius (A. Lutz, 1925)	Sfus	90
Scinax nasicus (Cope, 1862)	Snas	10
Scinax sp.	S_sp	25
Trachycephalus typhonius (Linnaeus, 1758)	Ttyp	2
**Leptodactylidae (11)**		
Adenomera sp.	A_sp	3
Leptodactylus elenae Heyer, 1978	Lele	2
Leptodactylus fuscus (Schneider, 1799)	Lfus	36

Leptodactylus macrosternum Miranda-Ribeiro, 1926	Lmac	48
Leptodactylus mystacinus (Burmeister, 1861)	Lmys	5
Leptodactylus podicipinus (Cope, 1862)	Lpod	500
Physalaemus albonotatus (Steindachner, 1864)	Palb	21
Physalaemus centralis Bokermann, 1962	Pcen	3
Physalaemus cuvieri Fitzinger, 1826	Pcuv	28
Physalaemus nattereri (Steindachner, 1863)	Pnat	2
Pseudopaludicola saltica (Cope, 1887)	Psal	14
**Microhylidae (Gastrophryninae) (2)**		
Dermatonotus muelleri (Boettger, 1885)	Dmue	1
Elachistocleis bicolor (Guérin-Méneville, 1838)	Ebic	5
**Phyllomedusidae (1)**		
Pithecopus azureus (Cope, 1862)	Pazu	1
**Total abundance**		**2162**
**Total richness**		**28**

Table S2. Anurans recorded at the São Vicente Institute, showing the abundance of each species at each pond (A-E). See Table S1 for full species names.

Species	Ponds				
	A	B	C	D	E
Rdip	24	3	2	2	4
Balb	13	0	0	0	0
Bpun	42	20	78	62	15
Bran	8	5	15	14	5
Deli	1	1	0	1	14
Dmin	70	31	3	147	43
Dnan	110	19	6	133	44
Ppla	18	3	0	103	3
Sacu	0	1	0	0	2
Sfmar	50	13	2	212	24
Sfus	2	15	42	8	23
Snas	0	0	8	0	2

S_sp	4	4	5	2	10
Ttyp	2	0	0	0	0
A_sp	0	1	2	0	0
Lele	0	1	0	0	1
Lfus	22	2	2	5	5
Lmac	12	2	17	10	7
Lmys	0	0	4	1	0
Lpod	152	61	142	77	68
Palb	4	2	1	2	12
Pcen	0	0	0	2	1
Pcuv	11	6	2	3	6
Pnat	1	1	0	0	0
Psal	14	0	0	0	0
Dmue	0	0	1	0	0
Ebic	1	1	1	2	0
Pazu	0	0	0	1	0
**Total abundance**	**561**	**192**	**333**	**787**	**289**
**Species richness**	**20**	**20**	**18**	**19**	**19**
